# Cystatin C Deficiency Increases LPS-Induced Sepsis and NLRP3 Inflammasome Activation in Mice

**DOI:** 10.3390/cells10082071

**Published:** 2021-08-12

**Authors:** Monika Biasizzo, Mojca Trstenjak-Prebanda, Klemen Dolinar, Sergej Pirkmajer, Janja Završnik, Boris Turk, Nataša Kopitar-Jerala

**Affiliations:** 1 Department of Biochemistry, Molecular and Structural Biology, Jožef Stefan Institute, SI-1000 Ljubljana, Slovenia; monika.biasizzo@ijs.si (M.B.); mojca.prebanda@ijs.si (M.T.-P.); janja.zavrsnik@gmail.com (J.Z.); boris.turk@ijs.si (B.T.); 2 International Postgraduate School Jožef Stefan, SI-1000 Ljubljana, Slovenia; 3 Institute of Pathophysiology, Faculty of Medicine, University of Ljubljana, SI-1000 Ljubljana, Slovenia; klemen.dolinar@mf.uni-lj.si (K.D.); sergej.pirkmajer@mf.uni-lj.si (S.P.); 4 Faculty of Chemistry and Chemical Technology, University of Ljubljana, SI-1000 Ljubljana, Slovenia; 5 Institute for Regenerative Medicine, Sechenov First Moscow State Medical University, 8-2 Trubetskaya st., 119991 Moscow, Russia

**Keywords:** Cystatin C, lipopolysaccharide, NLRP3 inflammasome, autophagy, cysteine proteases, interleukin-1 (IL-1)

## Abstract

Cystatin C is a potent cysteine protease inhibitor that plays an important role in various biological processes including cancer, cardiovascular diseases and neurodegenerative diseases. However, the role of CstC in inflammation is still unclear. In this study we demonstrated that cystatin C-deficient mice were significantly more sensitive to the lethal LPS-induced sepsis. We further showed increased caspase-11 gene expression and enhanced processing of pro-inflammatory cytokines IL-1β and IL-18 in CstC KO bone marrow-derived macrophages (BMDM) upon LPS and ATP stimulation. Pre-treatment of BMDMs with the cysteine cathepsin inhibitor E-64d did not reverse the effect of CstC deficiency on IL-1β processing and secretion, suggesting that the increased cysteine cathepsin activity determined in CstC KO BMDMs is not essential for NLRP3 inflammasome activation. The CstC deficiency had no effect on (mitochondrial) reactive oxygen species (ROS) generation, the MAPK signaling pathway or the secretion of anti-inflammatory cytokine IL-10. However, CstC-deficient BMDMs showed dysfunctional autophagy, as autophagy induction via mTOR and AMPK signaling pathways was suppressed and accumulation of SQSTM1/p62 indicated a reduced autophagic flux. Collectively, our study demonstrates that the excessive inflammatory response to the LPS-induced sepsis in CstC KO mice is dependent on increased caspase-11 expression and impaired autophagy, but is not associated with increased cysteine cathepsin activity.

## 1. Introduction

Sepsis is a life-threatening organ dysfunction caused by a dysregulated systemic response to infection [1]. Excessive or systemic exposure to lipopolysaccharide (LPS), a component of the Gram negative bacterial outer membrane, can cause a systemic inflammatory reaction leading to endotoxemia or sepsis [2]. A high-dose LPS injection into mice is a useful model for the study of endotoxic shock and associated inflammatory response. The recognition of LPS by Toll-like receptor 4 (TLR4) promotes inflammatory gene expression and pro-inflammatory cytokine production [3].

The NACHT, LRR and PYD domain-containing protein 3 (NLRP3) inflammasome is a multimeric protein complex comprising a sensor NLRP3, an adaptor protein apoptosis-associated speck-like protein (ASC) and an effector caspase-1 [4]. Activation of NLRP3 inflammasome is a two-step process that requires a priming step and activation. TLR4 signaling provides the priming signal that upregulates NLRP3 and interleukin (IL)-1β (IL-1β) precursor (pro-IL-1β) gene expression via nuclear factor kappa B (NF-κB)-associated pathways, the mitogen-activated protein kinase (MAPK) pathway and reactive oxygen species (ROS) [5,6]. The recognition of NLRP3 activators, such as extracellular ATP or bacterial toxins, leads to the assembly of the NLRP3 inflammasome, activation of caspase-1 and processing the pro-IL-1β to mature IL-1β [7]. Many different mechanisms have been proposed for inflammasome activation, such as K^+^ efflux, ROS overproduction, endoplasmic reticulum stress, mitochondrial dysfunction, Ca^2+^ signaling and lysosomal rupture [6,8]. Ion fluxes, including K^+^ efflux, Ca^2+^ mobilization and Cl^−^ efflux, have been proposed as crucial events in NLRP3 inflammasome activation. However, activation of caspase-1 can result in caspase-1-dependent inflammatory cell death, termed pyroptosis [9].

In addition to the canonical NLRP3 inflammasome, a non-canonical inflammasome pathway has been described [10]. Unlike caspase-1, caspase-11 (caspase-4/-5 in humans) does not require an upstream signaling cascade to detect LPS, but instead recognizes LPS directly in the cytosol [11,12]. Active caspase-11 can cleave and activate gasdermin D (GSDMD) directly, leading to pyroptotic cell death, but does not cleave IL-1β and IL-18 [11,12,13,14].

Autophagy, specifically macroautophagy, is an intracellular process important for cellular homeostasis and delivery of cytosolic constituents, including organelles, to lysosomes for degradation and amino acid recycling [15]. In the last decade, numerous studies have indicated that autophagy can regulate inflammasome activation, including NLRP3 inflammasome activation, through various mechanisms [16,17,18,19].

Cystatins are endogenous inhibitors of endolysosomal cysteine proteases, in particular cysteine cathepsins [20]. However, in the last two decades several cystatins have been suggested as involved in the immune system [21,22,23]. We have previously shown that stefin B, a type I cystatin that is the major intracellular cathepsin inhibitor, has a protective role in inflammation and endotoxemia [24,25]. The proposed model includes translocation of stefin B into mitochondria and protecting mitochondrial integrity upon LPS stimulation. In the absence of stefin B, increased mitochondrial ROS (mtROS) generation results in increased NF-κB activation and, consequently, caspase-11 expression [24]. Furthermore, stefin B-deficient BMDMs have decreased phosphorylation of ERK and p38 MAP-kinases, and have decreased IL-10 expression [25].

Cystatin C (CstC), on the other hand, is a type II cystatin that is the most abundant human cystatin found in the most body fluids [26]. Recent studies have demonstrated that cystatin C plays important roles in neuroprotection against various toxic stimuli via mechanisms such as inhibition of cysteine proteases, induction of cell division, induction of autophagy and anti-amyloidogenesis [21,27,28,29,30]. However, the role of cystatin C in inflammation and NLRP3 inflammasome activation is not yet clear.

In the present study, we show that cystatin C deficiency influences viability of mice in response to LPS and the NLRP3 inflammasome activation. Our findings highlight the immunomodulatory role of cystatin C in LPS-induced inflammation and NLRP3 inflammasome activation via induction of autophagy and regulation of signaling pathways rather than cysteine cathepsin inhibition.

## 2. Materials and Methods

### 2.1. General Reagents

A list of resources used in this study is provided in Table S1.

### 2.2. Mice

Cystatin C-deficient (CstC-/-) mice were established on a C57BL/6 strain in 1999 [31]. The CstC knockout (CstC KO) mice on C57BL/6 strain were kindly provided by Dr. Anders Grubb (Lund University, Lund, Sweden) and bred in our local colony. Mice (8–16 weeks of age) used in this study were wild-type (WT) and CstC KO, backcrossed for more than 10 generations to the FVB/N background.

### 2.3. Systemic LPS Challenge In Vivo

For the survival assay, WT and CstC KO mice were injected intraperitoneally (i.p.) with a high dose of LPS (*E. coli* 055:B5) at 30 mg/kg body weight and monitored six times daily over 96 h. For cytokine analysis, WT and CstC KO mice were injected intraperitoneally (i.p.) with a low dose of LPS at 5 mg/kg body weight for 4 or 24 h, as well as with PBS at 5 mL/kg body weight for 24 h to achieve the negative control. After LPS stimulation, mice were sacrificed and their vital organs were collected for further Western blot analysis.

### 2.4. Preparation of Macrophages

Mouse primary bone marrow-derived macrophages (BMDMs) were obtained by ex vivo differentiation from murine bone marrow progenitors flushed from femurs and tibiae in the presence of L929 medium over 7 days, as previously described [32]. During differentiation, cells were kept in DMEM supplemented with 20% L929 medium [32], 10% fetal bovine serum, 20 U of penicillin, 20 µg/mL of streptomycin, 2 mM L-glutamine, 1% MEM non-essential amino acids and 50 µM β-mercaptoethanol on non-treated 10-cm petri dishes in a humidified incubator with 5% CO_2_ at 37 °C.

### 2.5. qPCR

Total RNA was isolated from the BMDMs after stimulation with LPS (100 ng/mL) for 4 h in DMEM using PureLink RNA Mini Kit, followed by DNase treatment using a TURBO DNA-free Kit. RNA concentrations and A_260_/A_230_ and A_260_/A_280_ ratios were assessed spectrophotometrically using NanoDrop. cDNA was synthesized from RNA with a Precision nanoScript Reverse Transcription Kit using random nanomer primers. The primers designed by Primer Design (Table S2) were used for the quantitative real-time PCR. Real-time PCR was run on a Mx3005P qPCR system using the following thermal profile: 95 °C for 10 min, followed by 40 cycles of 95 °C for 15 s and 60 °C for 1 min. Additionally, a melting curve (55–95 °C) was performed at the end of each run. The mRNA expressions of target genes were normalized to the expression of *Gapdh* and *B2m*. The mRNA expression of genes was calculated considering their real time PCR efficiencies using REST 2009 (Relative Expression Software Tool) and presented as a fold increase, relative to the unstimulated cells (control). The mRNA expression levels of genes in control samples were normalized to 1.0.

### 2.6. Cell Lysate Preparation and Western Blot Analysis

BMDMs were prepared as described above, seeded in 6-well plates (2 × 10^6^ cells/well) and stimulated as indicated and described in Supplementary Methods. After the treatment, supernatants were collected and proteins precipitated with 10% TCA. Cells were washed with ice-cold PBS and cell lysates were prepared as previously described [25]. Protein concentration of the lysates were determined by a Bradford assay and equivalent amounts of protein from cell lysates were analyzed by SDS–polyacrylamide gel electrophoresis (SDS–PAGE) followed by Western blotting, as described previously [33]. The proteins were visualized with ECL according to the manufacturer’s instructions.

### 2.7. Cathepsin Activity Measurements

For cathepsin activity in cell lysates, BMDMs were seeded on 24-well plates (2 × 10^5^ cells/well); for extracellular cathepsin activity, BMDMs were seeded on 12-well plates (2 × 10^6^ cells/well) and stimulated as indicated and described in Supplementary Methods. After the treatment, the supernatants were collected and cells were washed with PBS; cathepsin activity was measured as described previously [34], using fluorogenic substrates Z-ArgArg-AMC (Z-RR-AMC) and Z-PheArg-AMC (Z-FR-AMC) at a final concentration of 30 µM. The liberation of AMC was measured using a fluorimeter and normalized on protein concentrations in cell lysates determined by a Bradford assay. Samples were further normalized to the mean value of the samples in the WT control group.

### 2.8. Cytokine Analysis

BMDMs were seeded on 96-well plates (10^5^ cells/well) and stimulated as indicated. After the treatment, the supernatants were collected and cells were washed with PBS and lysed with 2% Triton X-100. Concentration of IL-1β and IL-10 was determined by classical ELISA for IL-1β and ELISA for IL-10 following the manufacturer’s instruction. Nitric oxide production was determined by Griess reaction. Concentrations of cytokines and released NO were normalized to protein concentrations in cell lysates determined by Bradford assays.

### 2.9. Cytotoxicity, Lactate Dehydrogenase Release

BMDMs were seeded on 96-well plates (10^5^ cells/well) and stimulated as indicated. Release of the cytoplasmic enzyme lactate dehydrogenase (LDH) into the medium was determined by the cytotoxicity detection kit following the manufacturer’s instruction. BMDMs were lysed with 2% Triton X-100 to obtain total LDH release. The cytotoxicity was expressed as the percentage of the total LDH release.

### 2.10. Flow Cytometry

Lysosomal integrity was determined using LysoTracker Green. BMDMs were seeded on 12-well plates (4 × 10^5^ cells/well) and primed with LPS. Cells were washed with PBS, trypsinized for 10 min, harvested and pelleted at 1000× *g* for 5 min. Cells were loaded with LysoTracker Green (50 nM) in DMEM for 20 min and simultaneously stimulated with ATP (5 mM) for 20 min, as indicated. Cells were centrifuged at 1000× *g* for 5 min, immediately resuspended in PBS and subjected to FACS analysis according to the manufacturer.

Mitochondrial membrane potential (Δψ_m_) was determined using MitoTracker Red CMXRos (50 nM) and MitoTracker Green (50 nM). ROS-producing mitochondria were analyzed using MitoSOX Red (5 µM) and MitoTracker Green (50 nM). Cells were stimulated as indicated and loaded with corresponding fluorescent dyes in DMEM for 30 min. Cells were washed with PBS, trypsinized for 10 min, harvested, pelleted at 1000× *g* for 5 min, immediately resuspended in PBS and subjected to FACS analysis according to the manufacturer.

All samples were analyzed with a FACSCalibur flow cytometer, and fluorescence intensity plots and density plots were analyzed with FlowJo software. All flow cytometry analyses were performed following live cell gating from forward and side scatter profiles.

### 2.11. Analysis of OCR and ECAR with Seahorse XF Analyser

BMDMs were seeded in Seahorse XF24 cell culture microplates (2.2 × 10^5^ cells/well) in growth medium (DMEM with high glucose and 20% FBS). For the 12-h treatment: 36 h after seeding, cells were treated with LPS (100 ng/mL) or vehicle; 11 h later, growth medium was replaced with assay medium (Seahorse XF DMEM medium supplemented with glucose (10 mM), glutamine (2 mM) and pyruvate (1 mM)) with LPS (100 ng/mL) or vehicle. Cells were incubated for an additional 60 min at 37 °C without CO_2_ before a mito stress test. For the 4-h treatment: 48 h after seeding, cells were treated with LPS (100 ng/mL) or vehicle; 3 h later, growth medium was replaced with assay medium with LPS (100 ng/mL) or vehicle. Cells were incubated for an additional 60 min at 37 °C without CO_2_ before a mito stress test. The mito stress test was performed in Seahorse XFe24 Analyzer (Agilent) with oligomycin (1.5 μM), FCCP (1.5 μM) and rotenone (0.5 μM) + antimycin A (0.5 μM). After analysis in Seahorse, cells were lysed with 0.1% SDS in water and analyzed for protein content with BCA protein assay. All OCR and ECAR measurements were normalized to protein content before any further analysis.

### 2.12. Statistical Analysis

Average results are presented as the mean ± S.D. from the number of assays indicated. Statistical significance of the results was determined using an unpaired Student’s *t*-test. Survival rates were analyzed using the Kaplan–Meier method and compared using a log-rank (Mantel–Cox) test. OCR and ECAR were analyzed by two-way ANOVA using Bonferroni’s test.

## 3. Results

### 3.1. CstC KO Mice Are More Sensitive to LPS-Induced Lethal Endotoxemia and Produce Higher Levels of IL-18 in Spleen Post-LPS Administration

**Figure 1:**
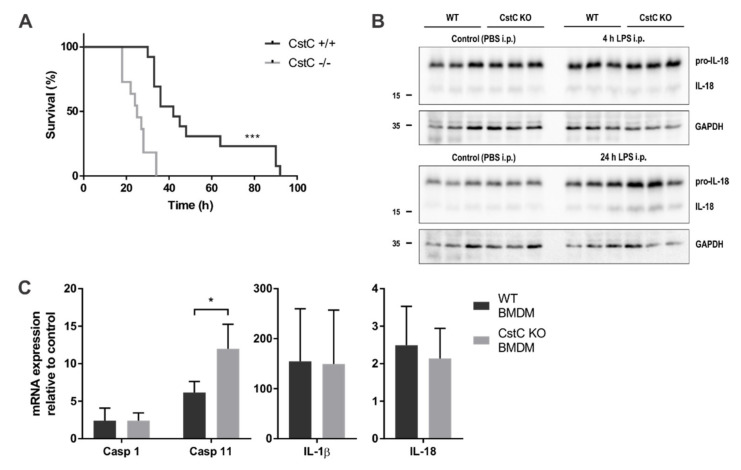
LPS-induced lethality and LPS-triggered pro-inflammatory cytokine and caspase expression is cystatin C-dependent. (**A**) Kaplan–Meier plot showing percentage of survival over time of age-matched wild-type (WT) (n = 13) and CstC KO mice (n = 11). *** *p* < 0.001 (log-rank (Mantel–Cox) test). Mice were injected intraperitoneally with 30 mg/kg LPS, and survival was monitored six times daily for a total of 4 days. (**B**) WT and CstC KO mice were injected with LPS at 5 mg/kg body weight for 4 h or 24 h and with PBS at 5 mL/kg body weight for 24 h for negative control. After LPS stimulation, mice were sacrificed and their spleens collected for Western blot analysis. Organ lysates were immunoblotted with indicated antibodies. Data shown are cropped blot images representative of three independent experiments. (**C**) BMDMs were stimulated with LPS (100 ng/mL) for 4 h and total RNA was isolated. Relative mRNA expression was determined, normalized to reference genes *Gapdh* and *B2m* and presented as fold increase compared to unstimulated (control) samples. Data were obtained from three independent experiments performed in triplicate, and the results are presented as means ± S.D. * *p* < 0.05, *** *p* < 0.001.

To investigate the importance and role of cystatin C in the LPS-induced lethal endotoxemia, mice were intraperitoneally injected with a high dose of LPS (30 mg/kg body weight) and survival was monitored over a 96-h period (Figure 1A). All CstC knock-out (KO) mice succumbed to the high dose of LPS within 34 h after challenge, whereas 70% of WT mice were still alive at 34 h after challenge and 25% of WT mice survived LPS-induced endotoxemia up to 92 h post-injection. 

Furthermore, we challenged CstC KO and WT mice (8–12 weeks of age) with a lower dose of LPS (5 mg/kg body weight) and analyzed spleen lysates with Western blot analysis (Figure 1B). Levels of pro-IL-18 and IL-18 in spleen lysates of CstC KO mice was increased 24 h post-LPS administration. These findings suggest that cystatin C deficiency results in the greater susceptibility to sepsis, which is at least partly due to the increase of pro-inflammatory cytokines.

To examine whether cystatin C affects pro-inflammatory cytokine and caspase expression, the relative mRNA expression levels of caspases-1 and -11, IL-1β and IL-18 were measured after LPS stimulation in BMDMs derived from CstC KO and WT mice (Figure 1C). In line with inducible caspase-11 and IL-1β gene expression [35,36,37], we detected an extensive upregulation of the caspase-11 and IL-1β gene expression in both WT and CstC KO BMDMs in response to LPS stimulation. Cystatin C deficiency had minimal or no effect on caspase-1, IL-1β and IL-18 mRNA expression, whereas relative caspase-11 gene expression was higher in CstC KO BMDMs.

### 3.2. Activity of Lysosomal Cathepsins Is Increased in CstC KO BMDMs upon NLRP3 Inflammasome Activation

**Figure 2:**
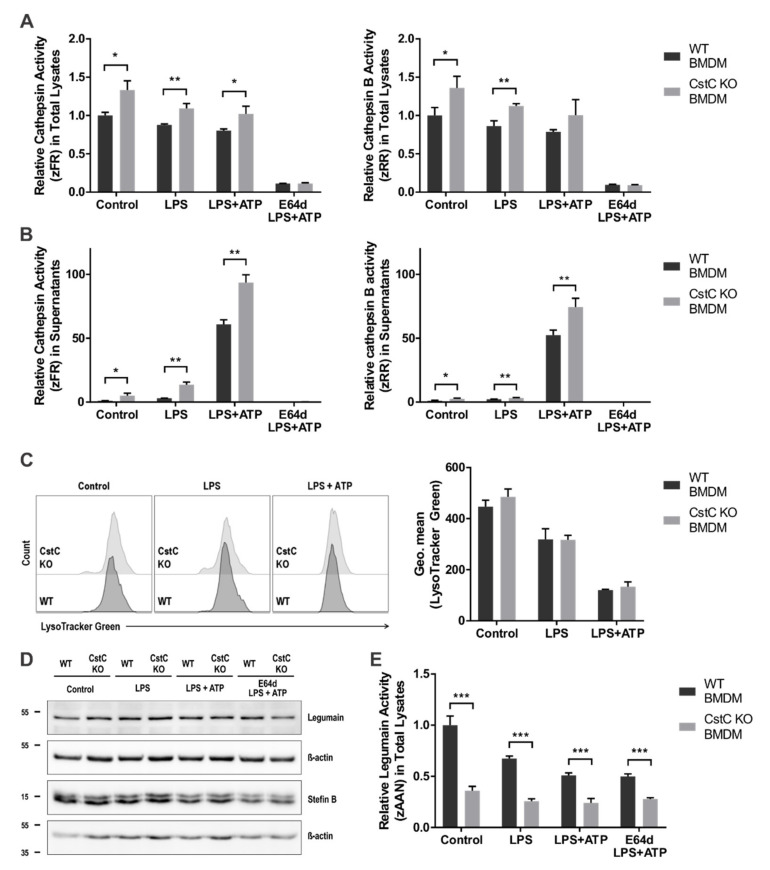
Increased cysteine cathepsin and decreased legumain activity in CstC KO BMDMs. BMDMs were pre-treated for 2 h with E-64d (20 µM), primed for 4 h with LPS (100 ng/mL) and stimulated for 20 min with ATP (5 mM) as indicated. (**A**) BMDMs were lysed with digitonin (200 µg/mL) and cysteine cathepsin activity was measured using fluorogenic substrates specific for cathepsins (zFR—cathepsin L-like activity; zRR—cathepsin B-like activity). (**B**) Supernatants were collected and cysteine cathepsin activity was measured using fluorogenic substrates specific for cathepsins. (**C**) Lysosomal integrity was analyzed in cells labelled with LysoTracker Green. (**D**) Cell lysates were immunoblotted with indicated antibodies. Data shown are cropped blot images, representative of three independent experiments. (**E**) BMDMs were lysed with digitonin (200 µg/mL) and legumain activity was measured using fluorogenic substrates specific for legumain (z-AAN). Data were obtained from three independent experiments performed in duplicate (panel **C**) or triplicate, and the results are presented as means ± S.D. * *p* < 0.05; ** *p* < 0.01; *** *p* < 0.001.

Since cystatin C is a potent cysteine cathepsin inhibitor, we hypothesized that cystatin C deficiency leads to increased cysteine cathepsin activity. We therefore investigated the effect of CstC deficiency on the activity of cysteine cathepsins upon the NLRP3 inflammasome activation by monitoring the hydrolysis of the fluorogenic broad cathepsin substrates Z-FR-AMC and Z-RR-AMC that are preferentially cleaved by cathepsins L and B, respectively. CstC KO and WT BMDMs were stimulated with LPS and ATP. In the total lysates we observed increased activity in the absence of cystatin C; however, after ATP treatment the difference in cathepsin B activity between the genotypes was no longer statistically significant (Figure 2A). In the supernatants, the activity was immensely increased after NLRP3 inflammasome activation and even further so in the absence of cystatin C (Figure 2B).

Previous studies showed lysosomal destabilization upon NLRP3 inflammasome activation with LPS/ATP stimulation, resulting in an increase of cysteine cathepsin activity in the cytosol [38,39]. Therefore, we examined lysosomal membrane integrity upon LPS and ATP stimulation with LysoTracker Green. The LPS priming slightly compromised lysosomal integrity, and the addition of ATP further increased lysosomal destabilization (Figure 2C). Nevertheless, CstC deficiency had no effect on the lysosomal integrity. In addition, we did not confirm any significant differences in the cathepsin activities between the genotypes in cytosolic cell extracts (Figure S1), confirming the above observation. Cysteine cathepsin activity additionally depends on the level of their other endogenous inhibitors, such as stefin B; however, we observed no differences in the amount of stefin B between CstC KO and WT BMDMs with Western blot (Figure 2D).

Since cystatin C is also an inhibitor of legumain, another lysosomal cysteine protease that is also known to be implicated in some inflammatory diseases [40], we next investigated the effect of CstC absence on legumain activity. We found that legumain activity upon LPS/ATP stimulation in total lysates of CstC KO BMDM was significantly reduced (Figure 2E). Legumain activity in BMDMs also depends on the level of legumain; however, we observed no differences in the amount of legumain between CstC KO and WT BMDMs with Western blot (Figure 2D). Contrary to our expectations, the legumain activity in CstC KO BMDMs decreased, indicating a lack of direct interaction between predominately secreted cystatin C and endolysosmal legumain. The mechanism by which a lack of cystatin C downregulates legumain activity is not yet understood. 

### 3.3. Increased Activation of NLPR3 Inflammasome in CstC KO BMDMs Is Independent of Cysteine Cathepsin Activity

**Figure 3:**
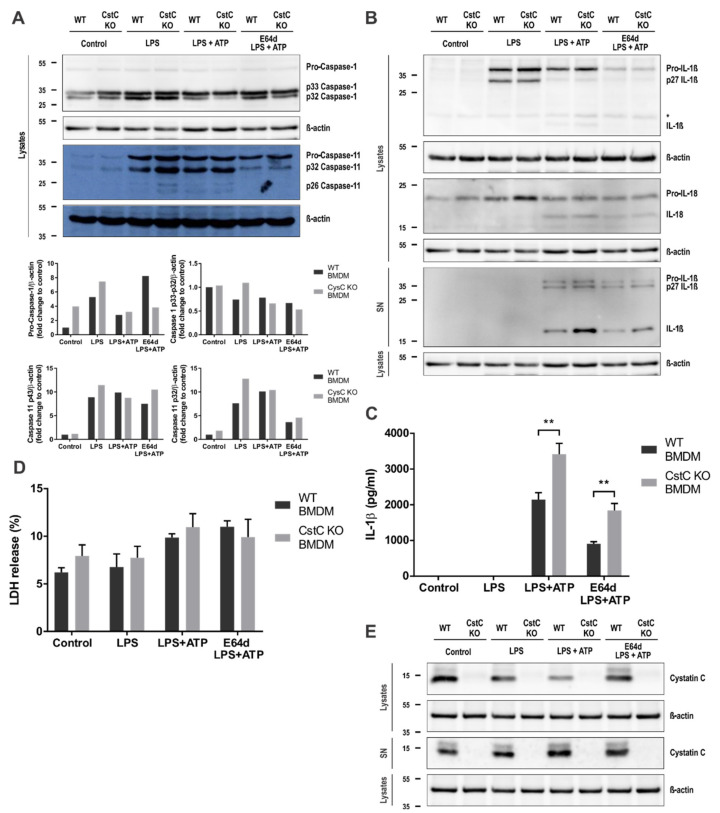
Increased activation of NLPR3 inflammasome in CstC KO BMDMs is independent of increased cysteine cathepsin activity. BMDMs were pre-treated for 2 h with E-64d (20 µM), primed for 4 h with LPS (100 ng/mL) and stimulated for 20 min with ATP (5 mM), as indicated. (**A**) and (**B**) Cell lysates were immunoblotted with indicated antibodies. Supernatants were precipitated and immunoblotted with indicated antibodies. (**C**) BMDMs were plated on 96-well plates and stimulated as described above. Supernatants were collected and IL-1β release was measured with enzyme-linked immunosorbent assay (ELISA). (**D**) Viability of BMDMs was assessed by LDH release into the cell culture media. The cytotoxicity was expressed as the percentage of the total LDH release. (**E**) Cell lysates were immunoblotted with indicated antibodies. Data shown are cropped blot images, representative of three independent experiments. Data were obtained from three independent experiments performed in triplicate, and the results are presented as means ± S.D. * *p* < 0.05; ** *p* < 0.01.

We further examined the effects of CstC deficiency on the activation of the NLRP3 inflammasome in BMDMs. Upon LPS priming and ATP stimulation, the formation of the p32 and p26 fragments of caspase-11 was more prominent in CstC KO BMDMs (Figure 3A), suggesting a more efficient self-cleavage and proteolytic activation of caspase-11 in these cells [41]. In line with the inducible IL-1β gene expression, pro-IL-1β in was detected in samples only after LPS priming. 

After the addition of ATP, the pro-inflammatory cytokines IL-1β and IL-18 were processed into their mature forms in both genotypes, in CstC KO BMDMs more prominently than in WT BMDMs (Figure 3B). The IL-1β mature form was detected by Western blot in cell lysates and in cell culture supernatants. Additionally, the increased IL-1β release in the cell culture supernatants of CstC KO BMDMs was determined by ELISA (Figure 3C).

To examine the role of increased cysteine cathepsin activity in NLRP3 inflammasome activation in CstC KO BMDMs, we pretreated BMDMs with the cell-permeable broad spectrum cathepsin inhibitor E-64d. However, even with complete inhibition of cysteine cathepsins (Figure 2A), the release of IL-1β in CstC-deficient BMDMs was significantly higher compared to the WT BMDMs (Figure 3C). Therefore, the increased cysteine cathepsin activity was not responsible for increased NLRP3 inflammasome activation in CstC KO BMDMs.

We examined the influence of CstC deficiency on NLRP3 protein expression; however there were no differences in NLRP3 expression in BMDMs upon LPS priming or inflammasome activation with ATP or SiO_2_ between the genotypes (Figure S2A,B). Nor did addition of cathepsin inhibitor E-64d or Ca-074me influence the expression of NLRP3. We determined the cleavage of GSDMD upon LPS/ATP stimulation and LPS/SiO_2_ stimulations in CstC KO and WT BMDMs lysates and cell culture supernatants. In CstC KO cell culture supernatants, we determined higher levels of cleaved p31 fragment of GSDMD (Figure S2A,B), indicating increased pro-inflammatory caspase activity in CstC KO BMDMs. Next, we investigated whether pyroptotic cell death is the cause of pro-inflammatory cytokine and cathepsin release. Therefore, we quantified the release of LDH in the cell culture media (Figure 3D and Figure S2C). However, the differences in LDH release were not significant between the genotypes after LPS/ATP stimulations (Figure S2C), nor upon LPS/SiO_2_ stimulations (Figure 3D). LDH was measured in CstC KO and WT BMDMs after LPS transfections with DOTAP (Figure S3B); however the differences were not significant between genotypes.

Since cystatin C is mostly extracellular protein, we next determined the levels of cystatin C in cell lysates and cell culture supernatants upon LPS/ATP stimulation. Intracellular cystatin C was found to be secreted from BMDMs after LPS priming and even further after ATP stimulation (Figure 3E). Secretion of cystatin C upon LPS/ATP stimulation from BMDMs was in line with the differences in cysteine cathepsin activities in cell lysates and supernatants between the two genotypes.

### 3.4. Cystatin C Deficiency Does Not Affect Mitochondrial Function or ROS Production

**Figure 4:**
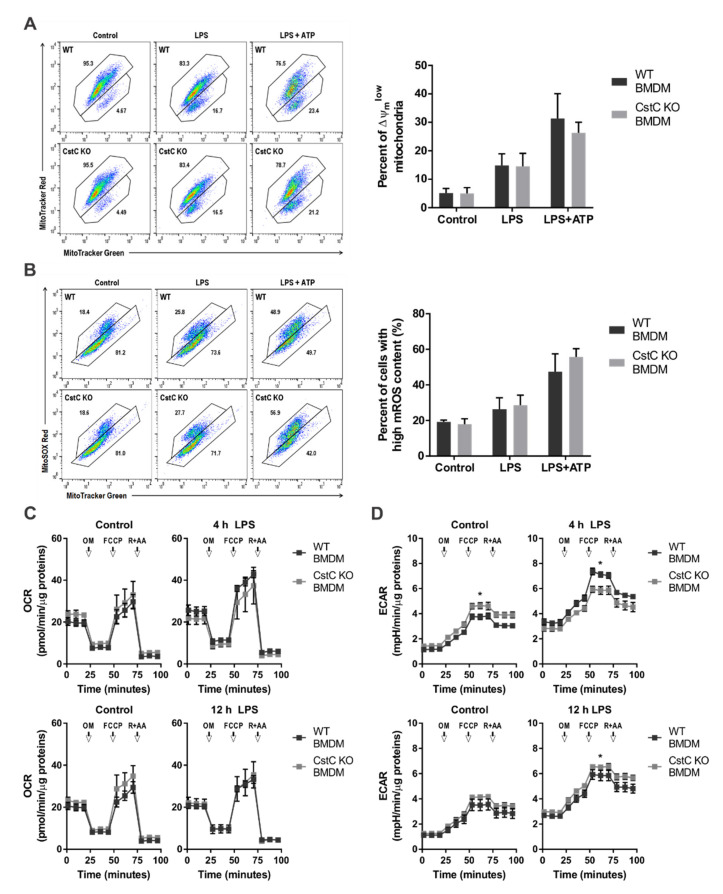
Cystatin C deficiency does not compromise mitochondrial membrane potential and generate mtROS. (**A**) and (**B**) BMDMs were primed for 4 h with LPS (100 ng/mL) and stimulated for 20 min with ATP (5 mM), as indicated. (**A**) Mitochondrial membrane potential (Δψm) was analyzed in cells labelled with MitoTracker Red CMXRos and MitoTracker Green. (**B**) ROS-producing mitochondria were analyzed in cells labelled with MitoSOX Red and MitoTracker Green. Data shown are representative of three independent experiments performed in duplicate, and the results are presented as means ± S.D. (**C**) and (**D**) BMDMs were stimulated with LPS (100 ng/mL) or vehicle (control) for 4 and 12 h. Mito stress test was then performed in Seahorse analyzer with oligomycin (OM) (1.5 μM), FCCP (1.5 μM) and rotenone + antimycin A (R+AA) (each 0.5 μM). Data shown are of three biological samples performed in single or duplicate, and the results are presented as means ± S.D. * *p* < 0.05 (two-way ANOVA with Bonferroni’s test).

Potentially, CstC could also affect ROS production. We therefore investigated the effect of CstC deficiency of mitochondrial dysfunction and ROS generation in BMDMs upon NLRP3 inflammasome activation. The loss of mitochondrial membrane potential (Δψ_m_) was monitored by flow cytometry. We stained BMDMs with MitoTracker Green for total mitochondrial content (Δψ_m_-independent mitochondrial stain) and MitoTracker Red CMXRos (Δψ_m_-dependent mitochondrial stain). The percentage of cells with dysfunctional mitochondria (MitoTracker Green^high^ and MitoTracker Red^low^ population) was increased after LPS priming and even further elevated following ATP stimulation; however, the differences between the genotypes were not statistically significant (Figure 4A).

Furthermore, we analyzed ROS-producing mitochondria using mitochondria-specific ROS indicator MitoSOX and MitoTracker Green. Stimulation of BMDMs with LPS/ATP increased the amount of ROS-producing mitochondria, more than in CstC KO BMDMs; however, the differences between the genotypes were not significant (Figure 4B).

In addition, we examined CstC-deficient BMDMs for changes in the rate of extracellular acidification (ECAR), and the mitochondrial rate of oxygen consumption (OCR) as a measure of glycolysis and oxidative phosphorylation (OXPHOS) after LPS stimulation. Furthermore, we analyzed the functional profile of mitochondria by determining real-time changes in OCR and ECAR during sequential treatment of cells with oligomycin (OM) (ATP synthase inhibitor), the cyanide p-trifluoromethoxyphenyl-hydrazone (FCCP) (H^+^ ionophore), and rotenone and antimycin A (inhibitors of the electron-transport chain). We did not determine any significant difference in the OCR between the genotypes (Figure 4C). However, CstC KO BMDMs had different dynamics of ECAR as WT BMDMs. In WT BMDMs, the ECAR substantially increased after 4 h LPS; however, the increase was minimal after 12 h LPS compared to controls. In CstC KO BMDMs the increase in ECAR after 4 h LPS was reduced compared to WT BMDMs, and remained increased after 12 h LPS (Figure 4D).

Stimulation of BMDMs with LPS and ATP led to greater ROS generation in both genotypes (Figure S4), whereas only LPS priming decreased oxidative stress, as determined with CM-H2DCFDA, a general oxidative stress indicator.

The above results suggest that mitochondrial (dys)function and ROS production are not CstC-dependent and thereby not the cause of increased NLRP3 inflammasome activation in CstC-deficient BMDMs.

### 3.5. Cystatin C Deficiency in BMDMs upon LPS Stimulation Does Not Affect MAPK Signalling Pathways, Anti-Inflammatory IL-10 Production and Nitric Oxide Production

**Figure 5:**
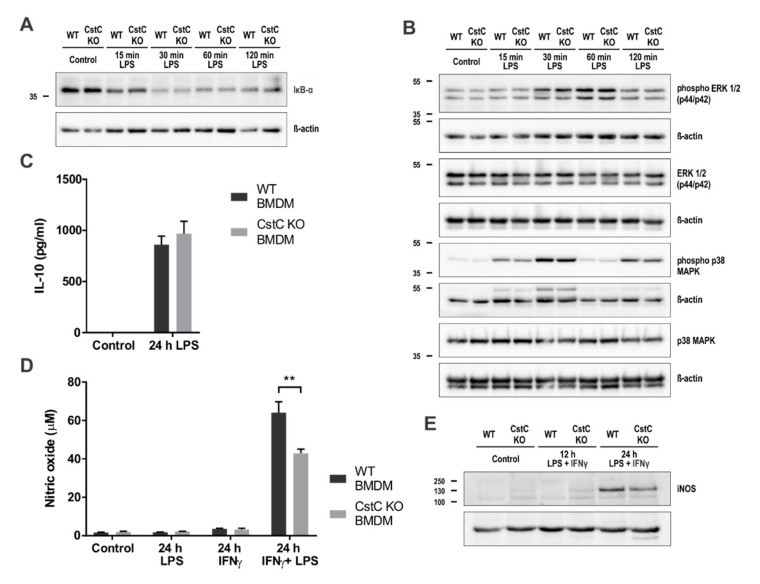
Cystatin C-deficiency in BMDMs upon LPS stimulation does not affect MAPK signaling pathways, anti-inflammatory IL-10 production or iNOS expression. (**A**) and (**B**) BMDMs were starved overnight and stimulated with LPS (100 ng/mL) for indicated times. Cell lysates were immunoblotted with indicated antibodies. (**C**) BMDMs were plated on 96-well plates and stimulated with LPS (100 ng/mL) for 24 h. Supernatants were collected and IL-10 release was measured with enzyme-linked immunosorbent assay (ELISA). (**D**) BMDMs were plated on 96-well plates and stimulated with LPS (100 ng/mL) or/and IFNγ (100 U/mL) for 24 h. Supernatants were collected and nitric oxide concentration measured with Griess reagent. (**E**) BMDMs were stimulated with LPS (100 ng/mL) and IFNγ (100 U/mL) for indicated times. Cell lysates were immunoblotted with indicated antibodies. Data shown are cropped blot images representative of three independent experiments. Data were obtained from three independent experiments performed in triplicate, and the results are presented as means ± S.D. ** *p* < 0.01.

Other potential pathways that could be affected are the NF-κB, MAPK and IL-10 signaling pathways, as well as NO production. We examined the effect of CstC deficiency on the activation of the NF-κB and MAPK (p38 and ERK) signaling pathways in BMDMs upon LPS stimulation by Western blot (Figure 5A,B). The protein level of the NF-κB inhibitor alpha (IκB-α) was diminished upon LPS stimulation, suggesting activation of the NF-κB signaling pathway in BMDMs (Figure 5A). The LPS stimulation induced MAPK phosphorylation and activation; however, the phosphorylation levels of investigated MAPKs were not affected by CstC deficiency (Figure 5B). To investigate whether CstC deficiency altered IL-10 secretion upon longer (24 h) LPS stimulation, we measured the IL-10 concentration in cell-culture supernatants after LPS stimulation in BMDMs with IL-10 ELISA. The secretion of anti-inflammatory cytokine IL-10 was comparable between CstC KO and WT BMDMs (Figure 5C).

In addition, the concentration of NO in cell culture supernatants after LPS and/or interferon gamma (IFNγ) stimulation in BMDMs was measured using Griess reagent (Figure 5D). Expression of iNOS in BMDMs after stimulation with LPS and IFNγ was determined by Western blot (Figure 5E). We observed a lower concentration of NO in cell culture supernatants of CstC KO BMDMs, as well as decreased iNOS protein levels in the cell lysates of CstC KO BMDMs.

### 3.6. CstC Ablation in BMDMs Decreases Autophagy Induction

**Figure 6:**
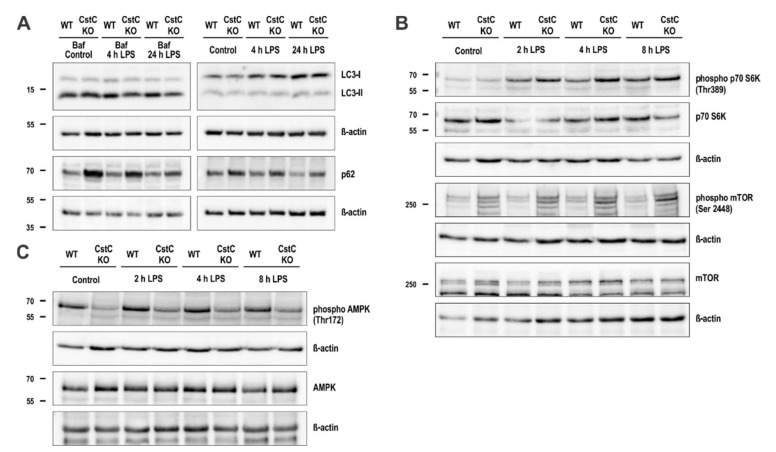
Decreased autophagy induction in CstC KO BMDMs. (**A**) BMDMs were pretreated with Bafilomycin A1 (80 nM) for 1 h, as indicated, and stimulated with LPS (100 ng/mL) for 4 or 24 h. (**B**) and (**C**) BMDMs were stimulated with LPS (100 ng/mL) for the indicated times. Cell lysates were immunoblotted with indicated antibodies. Data shown are cropped blot images representative of three independent experiments.

We examined the effect of CstC deficiency on autophagy after LPS stimulation in BMDMs by analyzing LC3 protein levels, a selective marker for autophagosomes, by Western blot (Figure 6A). In CstC KO BMDMs pre-treated with bafilomycin A1, an autophagy inhibitor, less LC3-II form was detected compared to the WT BMDMs, suggesting that CstC KO BMDMs had decreased numbers of autophagosomes. Correspondingly, the levels of SQSTM1/p62, an autophagy receptor, in CstC KO BMDM were increased.

In addition, we investigated the effect of CstC ablation on autophagy induction upon LPS stimulation by Western blot (Figure 6B,C). The mammalian target of rapamycin (mTOR) signaling pathway upon LPS stimulation in BMDMs was induced in both genotypes, but its induction was greater in CstC KO BMDMs, according to the phosphorylation level of mTOR substrate p70 S6K (Figure 6B). Additionally, phosphorylation level of 5′-AMP-activated protein kinase (AMPK) was decreased in CstC KO BMDMs (Figure 6C). 

Overall, the results above suggest that CstC KO BMDMs exhibit lower autophagy levels in normal conditions and upon LPS stimulation due to diminished autophagy initiation.

## 4. Discussion

This study provides new insight into the role of CstC in LPS-induced sepsis and NLRP3 inflammasome activation. We demonstrated that the lack of CstC resulted in increased lethality in LPS challenged mice, due to increased caspase 11 expression. Lee et al. [42] reported that caspase-11 auto-proteolysis is crucial for noncanonical inflammasome activation and GSDMD cleavage. GSDMD N-terminal fragment initiates pyroptosis as well as NLRP3 inflammasome activation of caspase 1 [13]. Therefore, the increased mortality upon LPS challenge in CstC-deficient mice could be attributed to the increased caspase-11 expression and consequently NLRP3 inflammasome activation [10]. Increased p31 GSDMD levels in cell culture supernatants of CstC KO BMDMs upon NLRP3 inflammasome activation with LPS/ATP and LPS/SiO_2_, as well as after LPS transfections, confirm increased activity of pro-inflammatory caspases 1 and 11 in CstC KO BMDMs. 

Several reports showed that an extracellular CstC could internalized in vivo [43], by human monocytes [44] and by various cancer lines [45,46]. Although an early study reported that the secretion of CstC was downregulated in murine peritoneal macrophages upon stimulation with LPS [47], in our experiments CstC was present in the cell media, as well as in cell lysates of WT BMDMs and was secreted upon LPS/ATP stimulation (Figure 3E). These differences could be due to the different amount of CstC internalization during different time points and cell type, as CstC is internalized via an active endocytic process [44]. The increased cysteine cathepsin activity in the cell media of CstC KO BMDMs confirms mostly extracellular function of CstC (Figure 2B). In our previous work, we showed increased cytosolic cysteine cathepsin activity upon LPS/ATP stimulation in BMDMs from stefin B-deficient mice [24]. However, in WT and CstC KO BMDMs the cytosolic activity of cysteine cathepsins was comparable between the two genotypes (Figure S1), confirming mostly extracellular inhibition of cysteine cathepsin by CstC. The effect of CstC deficiency on the processing and secretion of IL-1β was not reversed with complete inhibition of cysteine cathepsins by the E-64d inhibitor (Figure 3B,C), showing that cysteine proteinase activity is not essential for the differences in the inflammasome activation in CstC KO and WT BMDMs. Ca-074me inhibitor is quite specific for cathepsin B when used in vitro; however, when used in cell culture experiments it inhibits several lysosomal cathepsins. Therefore, it is not surprising that pre-treatment with Ca-074me inhibitor did not reverse better NLRP3 inflammasome activation in CstC KO BMDMs. In our previous work on stefin B-deficient BMDMs, incubation of BMDMs with the E-64d did not affect caspase-1 activation or secretion of IL-1β [24], indicating that the cathepsin activity is not crucial for NLRP3 inflammasome activation in stefin B-deficient mice [24]. Interestingly, a study using siRNA for stefin B and cystatin C reported upregulation of IL-1β transcript, not only IL-1β secretion upon SiO_2_ treated macrophages [48]. However, our results on BMDMs from stefin B-deficient mice [24] as well as our present study on CstC KO BMDMs have not confirmed these results. Since siRNA could activate innate RNA sensors, we cannot exclude the possibility that upregulation of the IL-1β or NLRP3 reported could be a consequence of an innate response to siRNA.

The levels of LDH in the cell media were relatively low even after LPS/ATP stimulation (<15%) (Figure 3D), and the differences between LDH release were not significantly different between CstC KO BMDMD and WT BMDMs also upon inflammasome activation with SiO_2_ or LPS transfections. The LPS-induced expression of caspase-11 was demonstrated to depend on the p38 MAPK/NF-κB signaling pathway [35,36,49]. Our results showed LPS-induced phosphorylation of p38 MAPK; however, there were no differences between CstC KO and WT BMDMs (Figure 5B). Correspondingly, IκB-α was significantly reduced upon LPS stimulation in both genotypes to the same amount (Figure 5A). Interestingly, Frendeus et al. reported that in IFNγ-stimulated macrophages, addition of CstC enhances NF-κB p65 activity [50]. Therefore, the effect of CstC on NF-κB activation is different from stefin B, where we reported impaired NF-κB activation upon increased expression [24]. 

In stefin B KO BMDMs we determined increased mtROS production and NF-κB activation that lead to increased caspase 11 expression. CstC deficiency had no effect on mitochondrial dysfunction, mtROS production or oxidative phosphorylation (Figure 4 and Figure S4); however, we determined different dynamics of ECAR, possibly indicating an increase in glycolysis in CstC KO BMDMs compared to WT controls upon LPS stimulations (Figure 4D).

Several studies have demonstrated that nematode cystatins [51], chicken cystatin [52,53] and human CstC [44] affect the MAPKs ERK and p38 pathways in different immune cells. Furthermore, we previously showed that stefin B-deficient BMDMs have decreased phosphorylation of ERK and p38 MAP-kinases, as well as decreased IL-10 expression [25]. The phosphorylation levels of investigated MAPKs (p38 and ERK1/2) upon LPS stimulation were unaffected by CstC deficiency (Figure 5A). The p38 MAPK was shown to at least partially mediate the expression of anti-inflammatory cytokine IL-10 [54,55], which can negatively regulate NLRP3 inflammasome [56]. In line with phosphorylation of p38 MAPK, IL-10 in cell culture supernatants after LPS stimulation in BMDMs was comparable between CstC KO and WT BMDMs (Figure 5C). Nematode cystatins and chicken cystatin were shown to induce the production of NO in IFN-γ stimulated macrophages [57,58], and further studies have revealed that CstC is involved in inducible nitric oxide synthase (iNOS) expression and NO production [50]. We determined a downregulation of NO production after IFNγ and LPS stimulation in CstC KO BMDMs (Figure 5D), as well as decreased iNOS protein levels in the cell lysates of CstC KO BMDMs (Figure 5E). Frendeus et al. reported that the lack of endogenous CstC in IFNγ-stimulated murine peritoneal macrophages did not influence NO production and iNOS mRNA expression; however, exogenously added CstC increased levels of iNOS and NF-κB activation [50]. The downregulation of NO production and iNOS expression upon IFNγ/LPS treatment in CstC KO BMDMs could contribute to lower NF-κB activation. 

CstC was shown to induce autophagy via suppression of mTOR activity or through AMP-activated kinase (AMPK) activation [30,59,60,61]. In our experiments, we determined lower levels of AMPK phosphorylation in CstC KO BMDMs. AMPK is also involved in regulation of the mTOR signaling pathway, with its phosphorylation suppressing mTOR activity [62].

Tizon et al. reported that the addition of CstC to neuroblastoma cells induced autophagy via the mTOR pathway [30]. mTOR kinase activity (mTORC1) was determined by the phosphorylation status of its downstream effector ribosomal protein S6 kinase beta-1 (p70 S6K). Stimulation of WT BMDMs with LPS resulted in mTORC1 activation, as indicated by increased phosphorylation of the downstream targets such as p70 S6K. In CstC KO BMDMs, we determined increased phosphorylation of the downstream targets of mTOR p70 S6K, when compared to WT BMDMs upon LPS stimulation (Figure 6B) and AMPK phosphorylation was suppressed in CstC KO BMDMs (Figure 6C), which is in line with previous studies reporting increased AMPK activation after the addition of CstC to cell cultures [30,60,61]. Autophagy removes damaged organelles, such as mitochondria, leading to reduced release of mitochondrial-derived DAMPs and mitochondrial ROS. CstC-deficient BMDMs show dysfunctional autophagy as the p62 is accumulated (Figure 6A). The higher levels of p62 could also result in impaired mitophagy. All these data combined indicate reduced autophagy flux in CstC-deficient BMDMs. Since autophagy and inflammation are tightly connected [18,19], the reduced autophagy flux could lead to an increased inflammatory response to NLRP3 inflammasome activation and LPS-induced sepsis in CstC-deficient BMDMs and mice.

The data presented here reveal a protective role of cystatin C in LPS-induced sepsis and NLRP3 inflammasome activation that is independent of protease inhibition. Our findings show that the lack of CstC resulted in impaired autophagy and dysregulated signaling pathways, which led to excessive inflammation. Therefore, cystatin C or its regulation may be an interesting candidate for treatment of disorders with excessive inflammation, such as sepsis, Crohn’s disease [44] or neurodegenerative disorders [30]. Regarding the important role of autophagy in inflammatory bowel diseases, CstC KO mice could serve as a good model to study the mechanisms of the disease.

Further investigation is necessary to elucidate the precise mechanisms of cystatin C modulate signaling pathways, such as autophagy induction and caspase-11 expression.

## Data Availability

All data generated or analyzed during this study are included in this published article (and its Supplementary Information Files).

## Supplementary Materials

The following are available online at https://www.mdpi.com/article/10.3390/cells10082071/s1. Table S1: List of resources used in this study. Table S2: Primers used for quantitative real-time PCR. Figure S1: Cytosolic cysteine cathepsin activity in CstC-independent in BMDMs upon NLRP3 inflammasome activation. Figure S2: NLRP3 priming and pyroptosis upon NLRP3 inflammasome activation with ATP or SiO_2_ crystals. Figure S3: Pyroptosis in BMDMs upon LPS transfection. Figure S4: Cystatin C deficiency has no effect of ROS production.
