# Supplementary material for: Cystatin C Deficiency Increases LPS-Induced Sepsis and NLRP3 Inflammasome Activation in Mice
Source: Cells. 2021 Aug 12;10(8):2071. doi: 10.3390/cells10082071 (PMC8391971; doi:10.3390/cells10082071)
Supplement: Supplementary file 1 [file cells-10-02071-s001.zip › cells-1295223-supplementary.pdf]

cells

MDPI

Supplementary Materials

Table S1. List of resources used in this study.

Reagent or resource	Source	Identifier
**Antibodies and recombinant proteins**		
mouse monoclonal anti-caspase-1	Novus Biologicals LLC, Centennial, CO, USA	Cat# NB100-56565
rat monoclonal anti-caspase-11	Abcam, Cambridge, MA, USA	Cat# ab10454
mouse monoclonal anti-IL-1β	National Cancer Institute, Frederick, MD, USA	3ZD
rabbit polyclonal anti-IL-18	Abcam, Cambridge, MA, USA	Cat# ab71495
mouse monoclonal anti-NLRP3	AdipoGen Life Sciences, San Diego, CA, USA	Cat# AG-20B-0014
rabbit monoclonal anti-GSDM D	Abcam, Cambridge, MA, USA	Cat# ab209845
rabbit polyclonal anti-stefin B	Abcam, Cambridge, MA, USA	Cat# ab53725
rabbit monoclonal anti-cystatin C	Abcam, Cambridge, MA, USA	Cat# ab109508
rabbit polyclonal anti-IκB-α	Cell Signalling Technology, Inc., Danvers, MA	Cat# 9242
rabbit polyclonal anti-phospho-p38 MAPK (T180/Y182)	Cell Signalling Technology, Inc., Danvers, MA	Cat# 9211
rabbit polyclonal anti-p38 MAPK	Cell Signalling Technology, Inc., Danvers, MA	Cat # 9212
mouse monoclonal anti-diphosphorylated ERK1/2	Sigma-Aldrich co., St. Louise, MO, USA	Cat# M8159
rabbit polyclonal anti ERK1/2	Abcam, Cambridge, MA, USA	Cat# ab17942
rabbit polyclonal anti-iNOS	Abcam, Cambridge, MA, USA	Cat# ab3523
rabbit polyclonal anti-mTOR phospho S2448	Invitrogen, Carlsbad, CA, USA	Cat# 441125G
rabbit polyclonal anti-mTOR	Cell Signalling Technology, Inc., Danvers, MA	Cat# 2972
rabbit monoclonal anti-p70 S6 kinase phospho T389	Cell Signalling Technology, Inc., Danvers, MA	Cat# 9234
rabbit monoclonal anti-p70 S6 kinase	Cell Signalling Technology, Inc., Danvers, MA	Cat# 2708
rabbit monoclonal anti-phospho-AMPKα (Thr172)	Cell Signalling Technology, Inc., Danvers, MA	Cat# 2535
mouse monoclonal anti-AMPKα1/2	Santa Cruz Biotechnology, Inc., Dallas, TX, USA	Cat# sc-74461
rabbit polyclonal anti-LC3B	Abcam, Cambridge, MA, USA	Cat# ab51520
mouse monoclonal anti-p62	Santa Cruz Biotechnology, Inc., Dallas, TX, USA	Cat# sc-48402
mouse monoclonal anti-	Sigma-Aldrich co.,	Cat# A1978

β-actin	St. Louise, MO, USA	
rabbit monoclonal anti-GAPDH	Cell Signalling Technology, Inc., Danvers, MA	Cat# 2118
rabbit monoclonal anti-legumain	Abcam, Cambridge, MA, USA	Cat# ab183028
	**Reagents, Buffers, and Solutions**	
DMEM	Sigma-Aldrich, St. Louise, MO, USA	Cat# D6429
FBS Heat-Inactivated	Sigma-Aldrich, St. Louise, MO, USA	Cat# F9665
DPBS	BioWest, Nuaillé, France	Cat# L0625
Penicillin-Streptomycin	Sigma-Aldrich, St. Louise, MO, USA	Cat# P4333
L-Glutamine solution	Sigma-Aldrich, St. Louise, MO, USA	Cat# G7513
MEM Non-Essential Amino Acids Solution (100X)	Gibco (Life Technologies), Paisley, UK	Cat# 11140035
D-(+)-glucose	Riedel-de-Haën	Cat# G8270
Sodium pyruvate solution	Sigma-Aldrich, St. Louise, MO, USA	Cat# S8636
β-mercaptoethanol	Sigma-Aldrich, St. Louise, MO, USA	Cat# 805740
OptiMEM	Invitrogen, Carlsbad, CA, USA	Cat# 31985-062
Tryple Select	Gibco (Life Technologies), Paisley, UK	Cat# 12563-029
LPS (E. coli 055:B5)	Sigma-Aldrich, St. Louise, MO, USA	Cat# L6529
ATP	Sigma-Aldrich, St. Louise, MO, USA	Cat# A6419
SiO_2 crystals	Invivogen, San Diego, CA, USA	Cat# tlrl-sio-2
Bafilomycin A1	Cayman Chemical, Ann Arbor, MI, USA	Cat# 11038
FCCP	Sigma-Aldrich, St. Louise, MO, USA	Cat# C2920
Antimycin A	Sigma-Aldrich, St. Louise, MO, USA	Cat# A8674
Oligomycin A	Merck Millipore, Burlington, MA, USA	Cat# 495455
Rotenone	Merck Millipore, Burlington, MA, USA	Cat# 557368
DOTAP	Roche Applied Science, Penzberg, Germany	Cat# 11202375001
E-64d	Peptide Institute, Osaka, Japan	Cat# 4321-v
Ca-074 me	MedChemExpress, Monmouth Junction, NJ, USA	Cat# HY-100350
z-FR-AMC	Bachem, Bubendorf, Switzerland	Cat# I-1160

z-RR-AMC	Bachem,<br>Bubendorf, Switzerland	Cat# I-1135
Digitonin	Sigma-Aldrich,<br>St. Louise, MO, USA	Cat# D141
Complete protease inhibitor mixture	Sigma-Aldrich,<br>St. Louise, MO, USA	Cat# P8340
Phosphatase arrest cocktail	G-Biosciences,<br>St. Louis, MO	Cat# 786-647
**Commercial assays**		
PureLink RNA Mini Kit	Ambion,<br>Austin, TX, USA	Cat# 12183018A
TURBO DNA-*free* Kit	Ambion,<br>Austin, TX, USA	Cat# AM1907
Precision nanoScript Reverse Transcription Kit	Primerdesign Ltd.,<br>Chandler's Ford, UK	Cat# RT-nanoScript
mouse geNorm Reference Gene Selection Kit	Primerdesign Ltd.,<br>Chandler's Ford, UK	Cat# ge-DD-12-mo
mouse IL-1 beta ELISA Ready-SET-Go!	eBioscience Technology,<br>San Diego, CA, United States	Cat# 88-7013-22
mouse IL-10 ELISA Ready-SET-Go!	Invitrogen,<br>Carlsbad, CA, USA	Cat# 88-7105-22
Cytotoxicity Detection KitPlus (LDH)	Roche Applied Science, Penzberg, Germany	Cat# 4744934001
**Equipment**		
NanoDrop 1000 Spectrophotometer	ThermoFisher Scientific,<br>Waltham, MA, USA	N/A
Mx3005P qPCR system	Agilent,<br>Santa Clara, CA, USA	N/A
G:BOX Chemi XR	Syngene,<br>Cambridge, UK	N/A
Tecan Infinite M1000 Pro	Tecan,<br>Gröding, Austria	N/A
FACSCalibur flow cytometer	Becton Dickinson,<br>Franklin Lakes, NJ, USA	N/A
**Software and algorithms**		
REST 2009 (Relative Expression Software Tool)	Technical University Munich, Munich, Germany<br>QIAGEN,<br>Hilden, Germany	N/A
GraphPad Prism	GraphPad Software,<br>San Diego, CA, USA	N/A
GeneSys	Syngene,<br>Cambridge, UK	N/A
CellQuest Pro	Becton Dickinson,<br>Franklin Lakes, NJ, USA	N/A
FlowJo v10	FlowJo LLC.,<br>Ashland, OR, USA	N/A

Table S2. Primers used for quantitative real-time PCR.

Protein (gene)	Forward Primer	Reverse Primer
IL-1β (*Il1b*)	GCTATGGCAACTGTT CCTGAA	ACAGCCCAGGTCAAA GGTT
IL-18 (*Il18*)	CCAAGTTCTCTTCGT TGACAAAA	GTCCTCTTACTTCACT GTCTTTG
Caspase-1 (*Casp1*)	CTGCGGTGTAGAAA AGAAACG	TCCATTTATTGTCCCT ATACTCACT
Caspase-11 (*Casp4*)	GCTACGATGTGGTG GTGAAA	GGAATGTGCTGTCTG ATGTCT

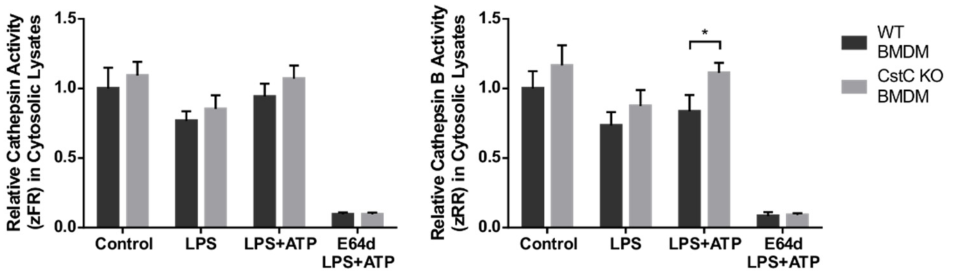

Figure S1. Cytosolic cysteine cathepsin activity in CstC-independent in BMDMs upon NLRP3 inflammasome activation. BMDMs were pre-treated for 2 h with E64d (20 μM), primed for 4 h with LPS (100 ng/ml) and stimulated for 20 min with ATP (5 mM), as indicated. BMDMs were lysed with digitonin (15 μg/ml) and cysteine cathepsin activity was measured using fluorogenic substrates specific for cathepsins (zFR – cathepsin L-like activity; zRR – cathepsin B-like activity). Data were obtained from three independent experiments performed in triplicate, and the results are presented as means ± S.D. *, $p < 0.05$.

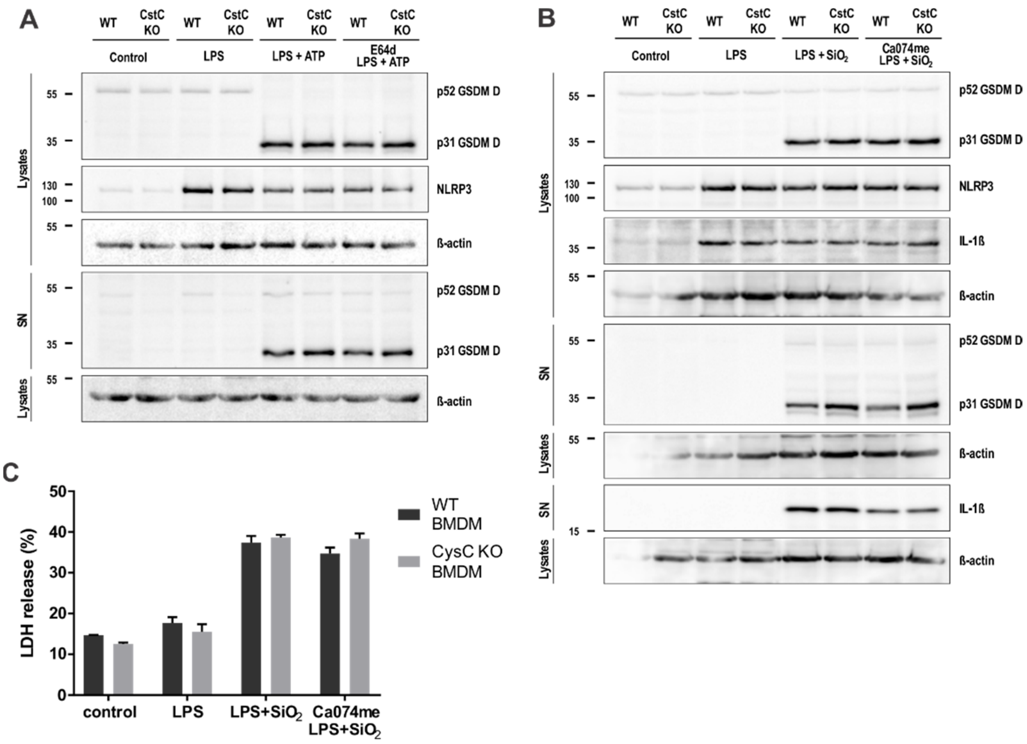

Figure S2. NLRP3 priming and pyroptosis upon NLRP3 inflammasome activation with ATP or SiO_2 crystals. *A*, BMDMs were pre-treated for 2 h with E-64d (20 μM), primed for 4 h with LPS (100 ng/ml) and stimulated for 20 min with ATP (5 mM) as indicated. *B*, BMDMs were pre-treated for 2

h with Ca-074 me (20 µM), primed for 4 h with LPS (100 ng/ml) and stimulated for 6 h with SiO_2 crystals (150 µg/ml). *A* and *B*, Cell lysates were immunoblotted with indicated antibodies. Supernatants were precipitated and immunoblotted with indicated antibodies. Data shown are cropped blot images representative of three independent experiments. *C*, BMDMs were plated on 96-well and stimulated as described above. Supernatants were collected and viability of BMDMs was assessed by LDH release into the cell culture media. The cytotoxicity was expressed as the percent of the total LDH release. Data were obtained from three independent experiments performed in triplicate, and the results are presented as means ± S.D.

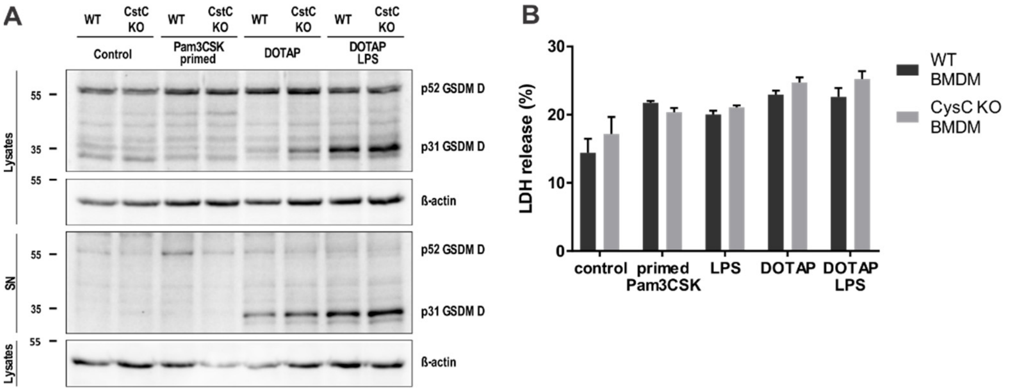

Figure S3. Pyroptosis in BMDMs upon LPS transfection. *A* and *B*, BMDMs were primed for 4 h with Pam3CSK (1 µg/ml) and transfected with DOTAP and LPS for 6 h, as indicated. *A*, Cell lysates were immunoblotted with indicated antibodies. Supernatants were precipitated and immunoblotted with indicated antibodies. Data shown are cropped blot images representative of three independependent experiments. *B*, BMDMs were plated on 96-well and stimulated as described above. Supernatants were collected and viability of BMDMs was assessed by LDH release into the cell culture media. The cytotoxicity was expressed as the percent of the total LDH release. Data were obtained from three independent experiments performed in triplicate, and the results are presented as means ± S.D.

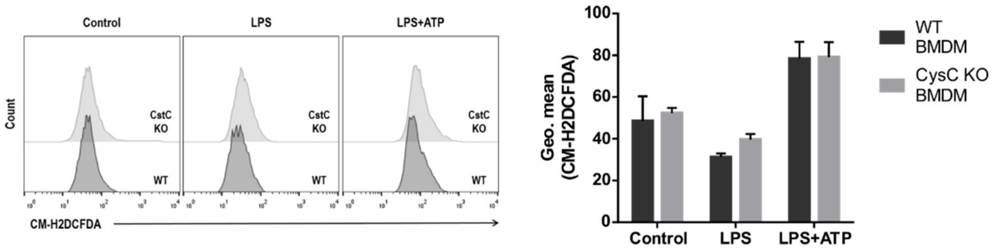

Figure S4. Cystatin C deficiency has no effect of ROS production. BMDMs were primed for 4 h with LPS (100 ng/ml) and stimulated for 20 min with ATP (5 mM). General oxidative stress and ROS generation was analysed in cells labelled with CM-H2DCFDA. Data shown are representative of three independent experiments performed in duplicate, and the results are presented as means ± S.D.
